# Visceral Adiposity and Anthropometric Indicators as Screening Tools of Metabolic Syndrome among Low Income Rural Adults in Xinjiang

**DOI:** 10.1038/srep36091

**Published:** 2016-10-26

**Authors:** Shu-xia Guo, Xiang-hui Zhang, Jing-yu Zhang, Jia He, Yi-zhong Yan, Jiao-long Ma, Ru-lin Ma, Heng Guo, La-ti Mu, Shu-gang Li, Qiang Niu, Dong-sheng Rui, Mei Zhang, Jia-ming Liu, Kui Wang, Shang-zhi Xu, Xiang Gao, Yu-song Ding

**Affiliations:** 1 Department of Public Health, Shihezi University School of Medicine, Shihezi, Xinjiang 832000, China; 2 Department of Pathology and Key Laboratory of Xinjiang Endemic and Ethnic Diseases (Ministry of Education), Shihezi University School of Medicine, Shihezi, Xinjiang 832000, China; 3 Department of Nutritional Sciences, The Pennsylvania State University 109 Chandlee Lab, University Park, PA 16801, USA.

## Abstract

Most previous studies on metabolic syndrome (MetS) examined urban and high income settings. We thus investigated the prevalence of MetS among a multi-ethnic population living in a low income rural area and explored the use of visceral adiposity and anthropometric indicators to identify men and women with MetS. We recruited 10,029 individuals of nomadic Kazakhs, rural Uyghur and Han residents in Xinjiang, China. MetS was defined by the Joint Interim Statement criteria. The receiver operating characteristic curve (ROC) was used to compare the area under the ROC curve (AUC) of each index. The age-adjusted prevalence of MetS was 21.8%. The visceral adiposity index (VAI), lipid accumulation product (LAP), body adiposity index (BAI) and the waist-to-height ratio (WHtR) were significantly associated with MetS, independent of ethnic, age, and other covariates. The AUC of VAI, LAP and WHtR were all greater than 0.7, and the LAP was the index that most accurately identified MetS status in men (AUC = 0.853) and women (AUC = 0.817), with the optimal cut-offs of 34.7 and 27.3, respectively. In conclusion, the prevalence of MetS in low income rural adults of Xinjiang was high and the LAP was an effective indicator for the screening of MetS.

Metabolic syndrome (MetS) is a cluster of metabolic abnormalities, characterized as dysglycemia, central obesity, raised blood pressure, elevated triglyceride (TG) levels, and low high-density lipoprotein cholesterol (HDL-C) levels^1^. MetS is associated with cardiovascular disease, type 2 diabetes morbidity and mortality, and all-cause mortality^2^. It is alarming that the prevalence of MetS is high and on the rise in both developing and developed nations^3^,^4^,^5^. Early identification and treatment of individuals with MetS could reduce risk of developing relevant diseases^6^. However, the unobvious physical problems or medical symptoms associated with this disorder make early identification of individuals with MetS challenging. Moreover, the cost and availability of diagnostic tests based on the MetS criteria have hindered the mass screening among high risk population, especially among underdeveloped areas and developing countries. Recently, several studies supported the use of waist-to-height ratio (WHtR) for the screening of MetS^7^,^8^,^9^, which is consistent with the notion that the metabolic complications of obesity could be more closely related to visceral adiposity than overall adiposity^10^,^11^,^12^. This is particularly important in Chinese population, who, despite being generally less obese, are more prone to visceral fat accumulation and insulin resistance compared with western populations^13^. However, the WHtR and waist circumference (WC), which is another commonly used surrogate marker of central adiposity and is included in the MetS diagnosis criteria, are inaccurate distinction between visceral and subcutaneous adipose tissue in the abdominal region.

Imaging techniques, such as magnetic resonance imaging and computed tomography, are considered the gold standard for quantitative evaluation of visceral adiposity, but the costs and radiation exposure associated with these two methods represent major barriers to their widespread use in clinical practice. In this context, it would be of interest to consider other visceral adiposity indicators which are feasible in general practice. The visceral adiposity index (VAI), a mathematical model that uses both anthropometric (BMI and WC) and metabolic (TG and HDL-C) parameters, and the lipid accumulation product (LAP), an index based on a combination of WC and TG, are two reliable markers of central lipid accumulation^14^,^15^. Further, the body adiposity index (BAI) is a composite index based on hip circumference and height, and was developed with the intention that this index would a direct estimate of percentage (%) body adiposity. It was thus suggested as an appropriate index of body adiposity by Bergman *et al*.^16^. Few studies, however, have examined whether the visceral adiposity indicators (VAI and LAP) and BAI perform better than WHtR for the screening of MetS, especially in a multi-ethnic population.

Xinjiang, a northwest province of China, contains 47 ethnic groups. However, the Uyghurs, Kazakhs and Hans accounted for 92.4% of the total population in Xinjiang. Moreover, most of the Xinjiang rural area is economically underdeveloped compared to other parts of China. It is crucial to seek cheap and effective parameters for the early screening of MetS and other cardiovascular risks. Thus, we conducted this study to examine the prevalence of MetS among a multi-ethnic population (Uyghurs, Kazakhs and Hans) living in Xinjiang rural area and explore the feasibility of visceral adiposity and anthropometric indicators (i.e., VAI, LAP, BAI and WHtR) for the screening of MetS. We also explored the optimal cut-off points of each index. Our observations may have important implications for the screening of MetS in other multi-ethnic populations, and provide basic data and theoretical evidence for the improvement of MetS criteria.

## Results

### The basic Characteristics of the Study Population

Compared to Non-MetS group (n = 7,464), MetS group (n = 2,565) had significantly higher levels of mean age, weight, BMI, WC, hip circumference, systolic BP, diastolic BP, TG, FBG, WHtR, VAI, LAP and BAI, and lower levels of height and HDL-C (*p* < 0.05 for all) (Table 1).

### Associations of visceral adiposity and anthropometric indices with MetS

The VAI, LAP, BAI and WHtR were strongly associated with the odds of having MetS in both men and women, after adjustment for potential confounders (*p for trend* < 0.001) (Table 2). ORs for MetS increased with increasing quartiles of all variables of interest. For all variables of interest, among both men and women, OR for MetS comparing two extreme quartiles was highest for LAP (39.7 for men and 23.0 for women), then followed by VAI (20.7 for men and 17.0 for women), WHtR (15.6 for men and 13.9 for women) and BAI (1.65 for men and 1.90 for women).

### Cut-off, sensitivity, specificity, Youden’s index and AUC of each variable for the screening of MetS using ROC Analyses

**Figure 1:**
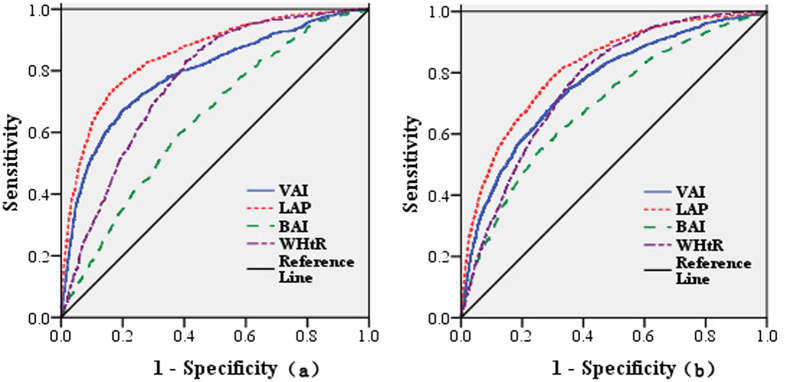
ROC curves of each variable for the screening of MetS in men and women according to the JIS criteria. (**a**) Men group; (**b**) Women group. Abbreviation: VAI: visceral adiposity index; LAP: lipid accumulation product; BAI: body adiposity index; WHtR: waist-to-height ratio.

According to the JIS criteria, in men, among all four visceral adiposity and anthropometric indicators, the LAP had the highest AUC value (AUC = 0.853), followed by VAI (AUC = 0.789), WHtR (AUC = 0.763) and BAI (AUC = 0.641). Correspondingly, LAP also had the highest Youden’s index of 0.571 (Sen = 73.93%, Spe = 83.15%), with the optimal cut-off as 34.7. The screening ability of LAP was followed by VAI (0.471), with the optimal cut-off as 1.71, and WHtR (0.422), with the optimal cut-off as 0.51, whereas BAI had the minimum Youden’s index (0.216). In women, the LAP still had the highest AUC value (AUC = 0.817), followed by WHtR (AUC = 0.764), VAI (AUC = 0.761) and BAI (AUC = 0.691). Similarly, LAP also had the highest Youden’s index of 0.486 (Sen = 78.88%, Spe = 69.71%), with the optimal cut-off as 27.3, followed by WHtR (0.414), with the optimal cut-off as 0.52, and VAI (0.395), with the optimal cut-off as 1.67, and the BAI still performed the minimum Youden’s index (0.290). In both men and women, the analysis that tested the statistical significance of the differences between AUCs revealed significant difference between AUCs for all pairs of all variables of interest, the exception is the pair of VAI and WHtR (Fig. 1 and Table 3).

As a secondary aim, we also examined the AVI, CI and WHR. The AUCs of AVI, CI and WHR were 0.775, 0.681 and 0.737 for men, and 0.769, 0.685 and 0.713 for women, respectively (Supplementary Fig. S1 and Supplementary Table S2).

## Discussion

In this large community-based and multi-ethnic population living in a low income rural area in the remote northwest region of China, we found that 21.8% of participants had MetS, based on the JIS criteria. Several visceral adiposity indicators, such as LAP and VAI appeared to be better indices to predict presence of MetS, relative to WHtR, which has been widely recommended based on previous studies^7^,^8^,^9^. To the best of our knowledge, this is the first large-scale population-based study to directly compare the screening ability of visceral adiposity indicators (LAP and VAI) and anthropometric indices (WHtR and BAI) for the screening of MetS in a multi-ethnic population. Our study presents evidence that the LAP and VAI may simple and useful tools for the screening of MetS among a multi-ethnic population in rural area.

There have been many epidemiology investigations on MetS, however, sparse data focused on poor and multi-ethnic areas due to reasons including, but not limit to, low public health priority, lack of expertise, inconvenient transportation and residence dispersal. These disparities lead to limited information for public health policy-making in low-income area. Our survey area was a typical low income and multi-ethnic area, 93% of whom lived on $1.00 or less, much higher than the national average^17^. According to the JIS criteria, the age-standardized prevalence of MetS in Xinjiang rural area was 21.8%. The prevalence was higher than the national level in China (16.5%)^5^and Japan (7.8%)^18^, whereas, lower than that in Spain (33.2%)^4^ and the US (34.3%)^3^. The possible reason may be that Xinjiang was a typical region for the diversity of cultures and ethnics, and was located on the “Silk Road” connecting the eastern world and the western world. The body fat status and dietary patterns are intertwined in Xinjiang, making the median prevalence of MetS between those of the Asian and European populations.

It is now widely acknowledged that, among the 5 components of MetS, increased adiposity is the most important because this measure is the core of the other 4 components^19^. Nevertheless, visceral adipose tissue adipocytes have a higher rate of lipolysis and also produce more adipocytokines, such as interleukin-6 and plasminogen activator inhibitor-1^20^. Several studies have suggested that visceral adiposity is almost well-validated for prediction of MetS^21^,^22^. Therefore, it is important to include routinely applicable indicators of visceral adiposity for the screening of MetS. In the present study, we found that there were significant relationships between VAI, LAP, WHtR as well as BAI and MetS in both men and women, the relationships independent of ethnic, age, smoking habits, alcohol consumption, education, marriage status and BMI. Furthermore, the risk of MetS as VAI, LAP as well as WHtR increased were significantly higher than that of BAI increased. This is explained by the fact that the variables making up the VAI, LAP, and WHtR are dichotomically expressed in the criteria for MetS. Interestingly, in our study, the ORs of VAI and LAP for the fourth quartiles in comparison with the first quartiles were extremely increased when compared with the other two indices, it may be due to the fact that WC, TG and HDL-C which make up the VAI and LAP are also included in the MetS diagnosis criteria. Individuals in the fourth quartiles of both VAI and LAP had higher levels of WC, TG and lower level of HDL-C than the cutoff points based on the MetS diagnosis criteria. Our study also found that associations between VAI/LAP/WHtR and MetS for the fourth quartiles were stronger in men than women, whereas the BAI was a stronger risk factor for MetS in women compared to men. Although explanations for these tissues remained to be elucidated, it is probably related to sex differences in regional adipose tissue distribution as well as patterns of visceral fat deposition^23^. Also, in fact, men have, on average, more visceral fat and less subcutaneous fat than women^24^.

Based on Hosmer and Lemeshow’s criteria, we found that LAP was an “excellent” indicator (AUC = 0.853 for men and AUC = 0.817 for women). Compared to the rest three indices, LAP was best able to discriminate individuals with and without MetS, which is consistent with previous studies conducted in Hans^25^ and Kazakhs^26^ of China. And study in Iran also showed the same conclusion^27^, suggesting that the LAP may be generally applied to people in China and other multi-ethnic countries. Both VAI and WHtR were “acceptable” indicators for the screening of MetS. Knowles *et al*.^28^ found that VAI was associated with all the components of MetS, furthermore, the three variables making up the VAI (WC, TG, and HDL-C) are all expressed in the criteria for MetS. However, in our study, the AUC of VAI for the screening of MetS was less than that of LAP in both men and women, which was opposite with a previous study^29^. Remarkably, the outcome variable used in their study was insulin resistance, not MetS. Furthermore, it is consider that the VAI is an indicator of early cardiometabolic risk in all borderline conditions in which overt MetS is not present^30^. This study purported that the utility of the BAI was “poor”. With validation studies consistently showing that the BAI tends to over or underestimate adiposity at extreme ends of percentage body fat, and that the BAI does not correlate with dual energy X-ray absorptiometer-derived% adiposity better than BMI when stratified by gender^31^. This may explain the poor utility for the screening of MetS in our study.

To date, there is not commonly accept cut-off point of LAP for the screening of MetS. In our study, the utility of LAP for the screening of MetS was demonstrated in the large community-based study with 10029 participants. More importantly, we determined optimal cut-off point for both men and women. According to the JIS criteria, the optimal cut-off values of LAP for the screening of MetS were 34.7 in men and 27.3 in women, which were much lower compared to those in Iran population (39.9 for men, 49.7 for women)^27^. It may be due to racial differences, which are caused by genetic and environmental exposure. However, our result was similar to those in Taiwanese people (31.6 for both men and women)^25^, suggesting that the LAP may be generally applied to Chinese people. The VAI is a sex-specific scoring system and has recently been suggested as a surrogated of visceral adiposity. However, there is no ideal cut-off value at which for the screening of MetS to date. Amato *et al*.^14^ assumed VAI = 1 in healthy individuals with normal adipose distribution and normal TG and HDL-C levels, lower than the cut-off values of VAI (1.71 in men and 1.67 in women) for the screening of MetS in our study. This could be due to that individuals who have an overt MetS are generally with higher TG and WC, and lower HDL-C levels. A systematic review suggested that WHtR greater than 0.5 may be a global boundary value for the screening of cardio-vascular disease^32^ which was supported by the simple message “limit your waist size to half of your height”^33^. However, in our study, the optimal cut-off values were higher (0.51 for men, 0.52 for women). This may be explained by the higher baseline level of obesity prevalence in far western China^34^.

Although the sensitivity of LAP for the screening of MetS was lower than that of WHtR, the Youden’s index of LAP was highest (0.571 for men, 0.486 for women) among all the indices of interest. As the fact that the higher of Youden’s index, the better validity for screening, the results of the present study demonstrated that the LAP may be a potential screening tool of MetS. Moreover, it should be pointed out that the sensitivity for the screening of MetS could be underestimated. Because adiposity is an essential status for MetS, the condition may lead to other metabolic disorders^35^.

With the increasing prevalence of MetS, this disorder has seriously burdened the society. Our findings thus have important public health and clinical implications. The rural communities we performed are typical low income settings, where the public resources are limited. For example, in Uyghur concentrated Jiashi county, more than 92% of Uyghurs live on $1.00 or less per day, relative to the national average (15.9%)^17^. Because MetS is prevalent among these low income rural areas, our study could help to develop a cost-effective screening strategy, which would not only benefit low income populations but also provide a good example of how to realize “good health at low cost” in developing countries^36^.

Our study has several limitations. First, it is a cross-sectional design, which precludes causal inferences. However the current study was designed for identifying simple and useful tools for the screening of MetS. Second, the VAI was established in Caucasian populations and the LAP was derived from studies of white, non-Hispanic blacks and Mexican Americans, and included the minimum sex-specific WC values of 65 cm for men and 58 cm for women, respectively, consistent with these criteria, individuals for whom the WC was less than this standard were not included in our study. The suitability for other populations need to be further investigated.

In conclusion, the prevalence of MetS in low income rural adults of Xinjiang was higher than the national level of China and fell in between the Euro-American and Asia levels. The LAP and VAI were both effective indicators for the screening of MetS, and the LAP was the most superior index to identify men and women with MetS, with the optimal cut-offs of 34.7 and 27.3 in women, respectively.

## Methods

### Ethics Statement

This study was approved by the Institutional Ethics Review Board (IERB) of the First Affiliated Hospital of Shihezi University School of Medicine (IERB No. SHZ2010LL01). All of the participants provided their written informed consent prior to the start of the study. Voluntary participation, anonymity and the confidentiality were guaranteed. All described methods were performed in accordance with the approved guidelines and regulations.

### Setting and Study Population

The survey was performed from 2009 to 2010 in Kashi (Uyghur), Yili (Kazakh) and Shihezi (Han) prefectures, respectively, which are approximately 2,739 miles from Beijing, and approximately 98% of the population are minority Muslim Uyghurs, Kazakhs or Hans. Multistage (prefecture-county-township-village) stratified cluster random sampling was employed to choose participants. First, we chose three representative prefectures (Kashi, Yili and Shihezi) based on the population, ethnicity, geography, economic and cultural development level, respectively in Xinjiang. Second, we randomly selected one county in each prefecture and one township from each county. Finally, a stratified sampling method was used to select the corresponding villages in each township. We interviewed local inhabitants aged ≥18 years who had resided in the village for at least 6 months and successfully interviewed a total of 10,029 (mean age 45.4 years; 3,122 Uyghurs, 3,694 Kazakhs and 3,213 Hans, respectively) individuals (who were interviewed to complete questionnaires, anthropometric measurements and blood tests). The overall response rate was 89.6% (91.5% for Uyghurs, 87.1% for Kazakhs and 90.3% for Hans, respectively).

### Assessment of MetS

WC and systolic and diastolic blood pressure were assessed by trained field workers using a standard protocol for participants during the interview and physical examination^37^. Blood samples were drawn from the cubital vein into tubes containing heparin sodium in the morning after an overnight fast. The detailed description of these methods has been published previously^38^. Serum glucose, TG and HDL-C concentrations, based on overnight fasting blood samples collected during the survey, were measured using a biochemical auto-analyzer (Olympus AU 2700; Olympus Diagnostics, Hamburg, Germany) in the clinical laboratory at the First Affiliated Hospital of Shihezi University School of Medicine.

In the current study, MetS was defined using the new Harmonized definition (JIS criteria)^39^, using specific WC cut-off points previously developed for the Chinese population (≥85 cm in men and ≥80 cm in women). Participants had to meet any 3 or more of the following 5 factors: WC ≥85 and 80 m for men and women, respectively, elevated TG level (>150 mg/dL or 1.70 mmol/L), reduced HDL-C (<40 mg/dL or 1 mmol/L in men, <50 mg/dL or 1.30 mmol/L in women), elevated systolic BP (≥130 mmHg) or diastolic BP (≥85 mmHg), and elevated FBG (≥100 mg/dL or 5.6 mmol/L).

### Assessment of visceral adiposity indicators

Body weight, height, hip circumference were measured during the survey, as detailed elsewhere^40^. Body Mass Index (BMI) was calculated as weight in kilograms divided by height in meters squared. WHtR was calculated by dividing the waist circumference by height. LAP was calculated as (WC (cm) − 65) × TG (mmol/L) for men, and (WC (cm) − 58) × TG (mmol/L) for women^15^. BAI was calculated as [hip circumference (cm)/(height (m))^1.5^ − 18]^16^. VAI was calculated using the following equation^14^:


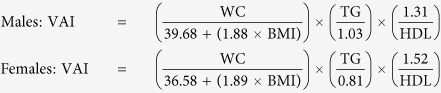


We also calculated other three indices (abdominal volume index (AVI), conicity index (CI) and waist-to-height ratio (WHR)) for abdominal adiposity^41^,^42^.

### Assessment of covariates

Detailed information on the participants was collected by a self-developed questionnaire according to face-to-face interview. The questionnaire included demographic, socioeconomic, dietary, medical history and lifestyles (e.g., smoking status).

### Statistical Analysis

Data were analyzed using SPSS version 17.0 for windows (SPSS Inc., Chicago, IL, USA). Continuous variables were presented as sex-specific mean ± standard deviation (M ± SD) and were analyzed using the t-test. Categorical variables were expressed as numbers or percentages and were analyzed using the Chi-square test. For each visceral adiposity and anthropometric indicator, we divided them into increasing sex-specific quartile values and used logistic regression analysis to calculate odds ratios (ORs) and 95% confidence intervals (CIs) for MetS across quartiles, with quartile 1 as reference group, adjusting for potentially confounding variables such as ethnic, age, smoking habits, alcohol consumption, education, marriage status and BMI.

The screening ability of each index to identify individuals with MetS was explored using receiver operating characteristic curve (ROC) analysis. Plots of sensitivity (true positives) versus 1 minus specificity (false positives) were constructed in both men and women for each of the parameters. The area under the curve (AUC) of the ROC and 95% confidence intervals (CIs) were used to determine which index showed the highest accuracy in screening MetS. The AUC is a measure of discrimination, and the AUC of 0.5, 0.6 ≤ AUC < 7, 7 ≤ AUC < 0.8, 0.8 ≤ AUC < 0.9 and ≥0.9 corresponded to no discrimination, poor, acceptable, excellent, and outstanding discrimination, respectively^43^, and the comparisons of ROC curves were performed using EmpowerStats statistical software (http://www.empowerstats.com). The optimal cut-off point of each of the parameters to identify individuals with MetS was defined by the maximum value of Youden’s index, which was calculated as sensitivity + specificity − 1. All statistical tests were two-sided, and differences were considered statistically significant at *p*-values < 0.05.

## Additional Information

**How to cite this article**: Guo, S.- *et al*. Visceral Adiposity and Anthropometric Indicators as Screening Tools of Metabolic Syndrome among Low Income Rural Adults in Xinjiang. *Sci. Rep.* **6**, 36091; doi: 10.1038/srep36091 (2016).

**Publisher’s note:** Springer Nature remains neutral with regard to jurisdictional claims in published maps and institutional affiliations.

## Supplementary Material

Supplementary Information

## Figures and Tables

**Table 1:** Basic characteristics of the study population.

Variables	MetS group (n = 2565)	Non-MetS group (n = 7464)	*t/χ2*	p-value*
	mean ± SD	mean ± SD		
Age (years)	51.1 ± 13.0	43.4 ± 14.3	−24.1	<0.001
Gender [n (%)]				
Men	1078 (42.0%)	3311 (44.4%)	4.22	0.040
Women	1487 (58.0%)	4153 (55.6%)		
Ethnic [n (%)]				
Uyghurs	682 (26.6%)	2440 (32.7%)	55.3	<0.001
Kazakhs	922 (35.9%)	2772 (37.1%)		
Hans	961 (37.5%)	2252 (30.2%)		
Height (cm)	161 ± 8.90	162 ± 8.51	2.39	0.017
Weight (kg)	67.5 ± 12.1	60.8 ± 10.9	−25.9	<0.001
BMI (kg/m^2^)	25.8 ± 3.74	23.1 ± 3.35	−33.8	<0.001
WC (cm)	90.8 ± 8.95	81.9 ± 9.73	−40.9	<0.001
hip circumference (cm)	99.8 ± 7.77	94.9 ± 7.30	−28.4	<0.001
Systolic BP (mmHg)	138 ± 22.1	122 ± 19.4	−35.4	<0.001
Diastolic BP (mmHg)	88.5 ± 13.2	78.5 ± 12.3	−34.8	<0.001
TG (mmol/L)	1.71 ± 0.77	1.09 ± 0.58	−42.6	<0.001
HDL-C (mmol/L)	1.32 ± 0.62	1.43 ± 0.58	8.46	<0.001
FBG (mmol/L)	5.59 ± 1.87	4.65 ± 0.93	−33.1	<0.001
WHtR	0.56 ± 0.06	0.51 ± 0.06	−41.5	<0.001
VAI	2.40 ± 1.21	1.35 ± 0.83	−48.7	<0.001
LAP	49.8 ± 24.5	22.7 ± 15.9	−64.0	<0.001
BAI	30.8 ± 5.14	28.2 ± 4.43	−24.7	<0.001

Note: ^*^p-difference between MetS group and Non-MetS group. Abbreviation: BMI: body mass index; WC: waist circumference; TG: triglycerides; HDL-C: high-density lipoprotein cholesterol; FBG: fasting blood glucose; WHtR: waist-to-height ratio; WHR: waist-to-hip ratio; VAI: visceral adiposity index; LAP: lipid accumulation product; BAI: body adiposity index.

**Table 2:** Adjusted odds ratios and 95%CI of MetS according to levels of VAI, LAP, BAI, and WHtR, respectively.

Variables	OR (95%CI)			
	Q1	Q2	Q3	Q4
**Men**				
VAI	Ref	1.34 (0.91–1.99)	3.19 (2.25–4.54)	20.7 (14.6–29.0)
LAP	Ref	3.09 (1.98–4.81)	6.50 (4.22–10.0)	39.7 (25.7–61.4)
BAI	Ref	1.28 (1.01–1.64)	1.55 (1.30–2.11)	1.65 (1.27–2.14)
WHtR	Ref	4.36 (2.76–6.88)	11.3 (7.17–17.7)	15.6 (9.74–24.9)
**Women**				
VAI	Ref	1.63 (1.19–2.25)	4.09 (3.02–5.55)	17.0 (12.5–23.1)
LAP	Ref	3.08 (2.12–4.47)	6.46 (4.49–9.29)	23.0 (15.9–33.2)
BAI	Ref	1.21 (0.97–1.51)	1.34 (1.07–1.67)	1.90 (1.50–2.42)
WHtR	Ref	4.21 (2.71–6.54)	9.11 (5.92–14.0)	13.9 (8.91–21.8)

Note: data was adjusted for ethnic, age, smoking habits, alcohol consumption, education, marriage status and BMI. CI-confidence interval. Abbreviation: VAI: visceral adiposity index; LAP: lipid accumulation product; BAI: body adiposity index; WHtR: waist-to-height ratio.

**Table 3:** The cut-off, Sen, Spe, Youden’s index and AUC of each variable for the screening of MetS in men and women.

Variables	cut-off	Sen %	Spe %	Youden’s index	AUC (95%CI)	p-value*
**Men**						
VAI	1.71	66.79	80.28	0.471	0.789 (0.772–0.805)	0.230
LAP	34.7	73.93	83.15	**0.571**	**0.853 (0.840–0.867)**	<0.001
BAI	27.1	57.51	64.11	0.216	0.641 (0.623–0.659)	<0.001
WHtR	0.51	86.64	55.54	0.422	0.763 (0.748–0.778)	Ref
**Women**						
VAI	1.67	69.40	70.09	0.395	0.761 (0.747–0.775)	0.820
LAP	27.3	78.88	69.71	**0.486**	**0.817 (0.804–0.829)**	<0.001
BAI	32.1	54.74	74.21	0.290	0.691 (0.676–0.707)	<0.001
WHtR	0.52	82.58	58.78	0.414	0.764 (0.751–0.777)	Ref

Note: ^*^p-difference between AUCs compared with WHtR. variables with highest Youden’s index and highest AUC value in **bold.** Abbreviation: Sen-sensitivity; Spe-specificity; VAI: visceral adiposity index; LAP: lipid accumulation product; BAI: body adiposity index; WHtR: waist-to-height ratio.
