# Supplementary material for: Visceral Adiposity and Anthropometric Indicators as Screening Tools of Metabolic Syndrome among Low Income Rural Adults in Xinjiang
Source: Sci Rep. 2016 Oct 26;6:36091. doi: 10.1038/srep36091 (PMC5080571; doi:10.1038/srep36091)
Supplement: Supplementary Information [file srep36091-s1.pdf]

Visceral Adiposity and Anthropometric Indicators as Screening Tools of Metabolic Syndrome among Low Income Rural Adults in Xinjiang

Shu-xia Guo, Xiang-hui Zhang, Jing-yu Zhang, Jia He, Yi-zhong Yan, Jiao-long Ma, Ru-lin Ma, Heng Guo, La-ti Mu, Shu-gang Li, Qiang Niu, Dong-sheng Rui, Mei Zhang, Jia-ming Liu, Kui Wang, Shang-zhi Xu, Xiang Gao, Yu-song Ding

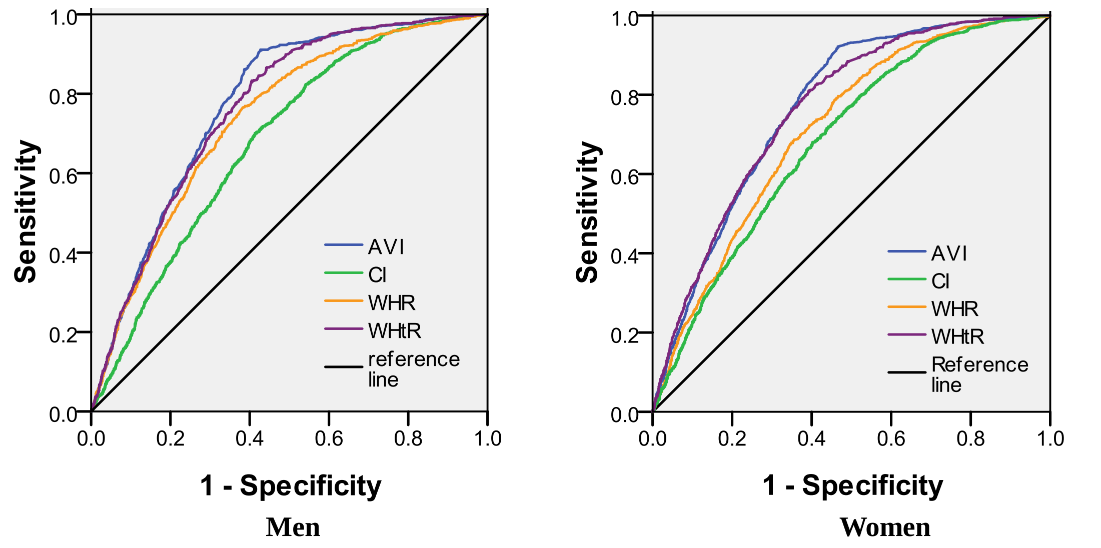

Supplementary Fig. S1. ROC curves of AVI, CI, WHR and WHtR for the screening of MetS in men and women according to the JIS criteria.

Abbreviation: AVI: abdominal volume index CI: conicity index; WHR: waist to-hip ratio; WHtR: waist-to-height ratio

Supplementary Table S2. AUC of AVI, CI, WHR and WHtR and 95% CI for the screening of MetS in men and women

Variables	Men			Women		
	**AUC**	**95%CI**	***p***	**AUC**	**95%CI**	***p***
AVI	0.775	(0.760-0.789)	>0.05	0.769	(0.756-0.782)	>0.05
CI	0.681	(0.664-0.698)	<0.05	0.685	(0.671-0.700)	<0.05
WHR	0.737	(0.721-0.754)	<0.05	0.713	(0.699-0.727)	<0.05
WHtR	0.763	(0.748-0.778)	Ref	0.764	(0.751-0.777)	Ref

Abbreviation: AUC: area under the curve; AVI: abdominal volume index CI: conicity index; WHR: waist to-hip ratio; WHtR: waist-to-height ratio
